# Deciphering the triad of endothelial glycocalyx, von Willebrand Factor, and P-selectin in inflammation-induced coagulation

**DOI:** 10.3389/fcell.2024.1372355

**Published:** 2024-04-30

**Authors:** Guinevere Ferreira, Alexandra Taylor, Solomon A. Mensah

**Affiliations:** ^1^ Biomedical Engineering Department, Worcester Polytechnic Institute, Worcester, MA, United States; ^2^ Mechanical Engineering Department, Worcester Polytechnic Institute, Worcester, MA, United States

**Keywords:** von Willebrand Factor, endothelial glycocalyx, P-selectin, heparan sulfate, coagulation

## Abstract

This review examines the endothelial glycocalyx’s role in inflammation and explores its involvement in coagulation. The glycocalyx, composed of proteins and glycosaminoglycans, interacts with von Willebrand Factor and could play a crucial role in anchoring it to the endothelium. In inflammatory conditions, glycocalyx degradation may leave P-selectin as the only attachment point for von Willebrand Factor, potentially leading to uncontrolled release of ultralong von Willebrand Factor in the bulk flow in a shear stress-dependent manner. Identifying specific glycocalyx glycosaminoglycan interactions with von Willebrand Factor and P-selectin can offer insights into unexplored coagulation mechanisms.

## 1 Introduction

The endothelial glycocalyx constitutes a dense layer of carbohydrate-rich structures that lines the endothelium (Reitsma et al., 2007; Möckl, 2020). It plays a crucial role in transducing mechanical forces within endothelial cells (EC) and regulates various vascular physiological activities. Despite its significance, many aspects of the glycocalyx functions remain unknown, particularly its involvement in coagulation and the tethering of clotting Factors. During inflammatory states, such as SARS-CoV-2 infections, there is documented degradation of the glycocalyx (Lipowsky et al., 2011; Zha et al., 2022). The glycocalyx is suggested to engage with clotting Factors, including von Willebrand Factor, a key player in platelet aggregation to stabilize injury sites (Pipe et al., 2016). These interactions create a delicate equilibrium, ensuring the cessation of bleeding while preventing the excessive formation of clots in the bloodstream. This review explores the intricate connections between the glycocalyx, its associated glycosaminoglycans (GAGs), von Willebrand Factor, and P-selectin in the context of inflammatory pathologies.

## 2 The endothelium

### 2.1 The endothelial glycocalyx

**FIGURE 1:**
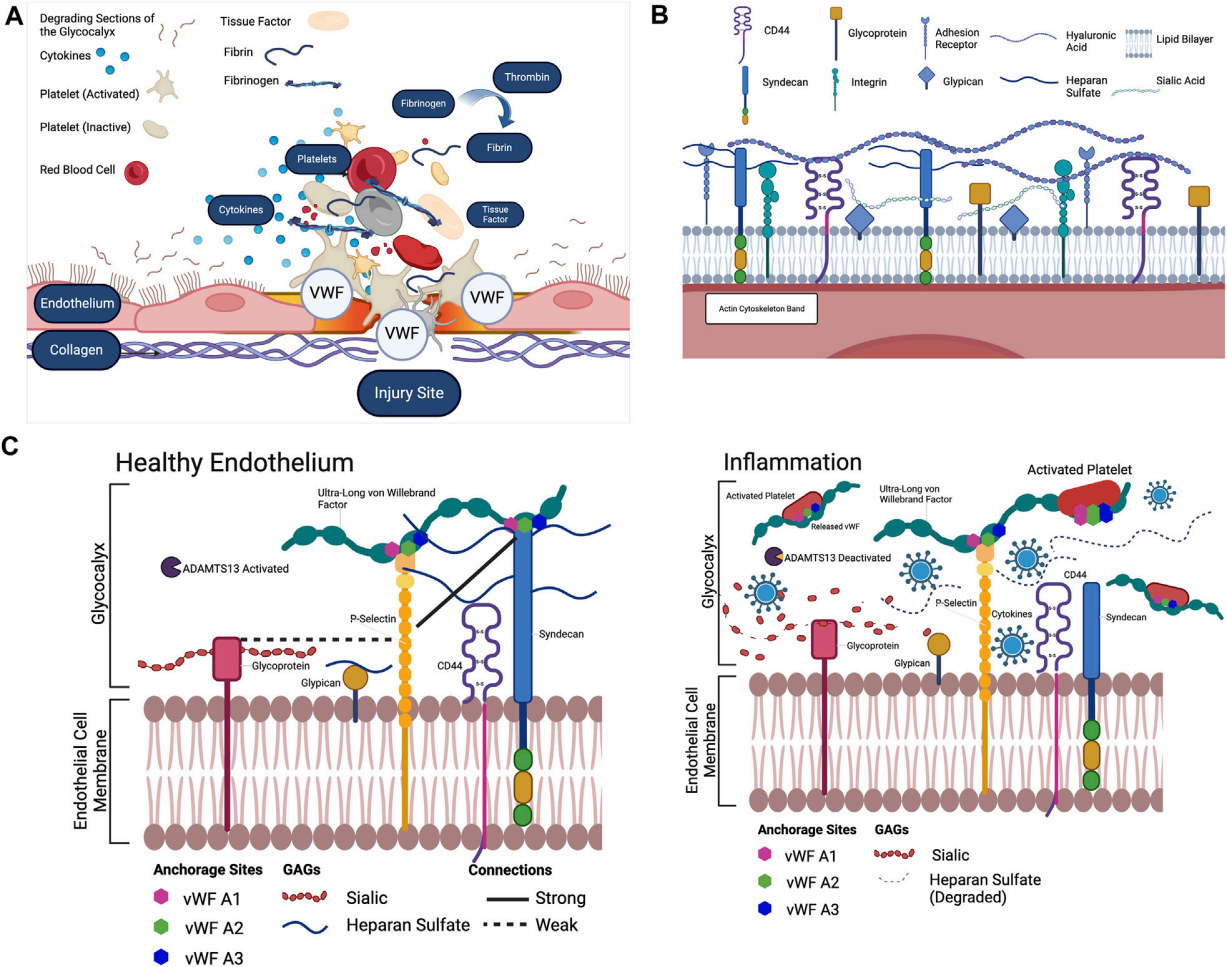
**(A)** The extrinsic pathway begins with Tissue Factor. Tissue Factor is primarily expressed in the walls of blood vessels. When the vessel wall is damaged, a large amount of Tissue Factor enters the blood. Tissue Factor combines with present proconvertin (Factor VII) to form the activated complex TF: FVIIa (Owens and Mackman, 2010; Neubauer and Zieger, 2022). TF: FVIIa activates Stuart-Power Factor (Factor X) and Factor VIII (O’Donnell et al., 2019). The extrinsic pathway can be terminated by tissue Factor pathway inhibitor, which inhibits the TF: FVIIa complex (Owens and Mackman, 2010). The intrinsic pathway begins with high molecular weight kininogen. Factor XV activates Hageman Factor. Factor XIIa combines with coFactors to activate Factor XI (Palta et al., 2014). Factor XI activates Factor IX. Factor IX activates Factor VIII. Factor IX and Factor VIII form a complex on the platelet phospholipids that activates Factor X (Owens and Mackman, 2010). In the common pathway, activated Stuart-Prower Factor (Factor Xa) and Factor Va form a prothrombinase complex with platelet phospholipids. Prothrombinase cleaves Prothrombin (Factor II) into Thrombin. Thrombin cleaves Fibrinogen (Factor I) into Fibrin (Owens 3rd and Mackman, 2010). Thrombin activates Factor XIII. Factor XIII crosslinks Fibrin into a polymer with covalent bonds. The platelets and Fibrin polymers form the haemostatic clot (Palta et al., 2014). To position the clot in the correct location, Von Willebrand Factor (Factor XVI) binds to collagen exposed by the injury to the vessel epithelium (Neubauer and Zieger, 2022). The Von Willebrand Factor then binds to the GPIb-IX-V complex on the platelet membrane, positioning the platelets and fibrin of the clot at the site of the injury (Sang et al., 2021). In recent years, the glycocalyx has been proposed to potentially play an important role in the anchoring of clotting Factors, and its well documented degradation in inflammatory states has been proposed as an underlying Factor for excessive clot development. Created with BioRender.com. **(B)** The endothelial glycocalyx and the GAGs that are present in the endothelial glycocalyx. Heparan sulfate, attached to the syndecan complex, hyaluronic acid and sialic acid and the glypican complex are the main components of the glycocalyx, which lines the endothelium (Mensah et al., 2021). Created with BioRender.com. **(A–C)** The endothelial glycocalyx during both inflammatory and healthy conditions. As shown, when cytokines are present to degrade sialic acid and heparan sulfate, the only known component anchoring the ULVWF to the endothelial membrane is P-Selectin, however much is unknown about the interactions of Sialic Acid, Heparan Sulfate and Hyaluronic Acid separately as anchors to the ULVWF. Each component must be separately stained to visualize and uncover these interactions in inflammatory conditions (Mensah et al., 2021). Created with BioRender.com.

Endothelial cells form the inner lining of blood vessels (Alberts et al., 2002). The endothelial glycocalyx is located at the apical side of these cells and it’s composed of proteins and carbohydrates responsible for regulating extracellular functions, such as cell signaling transduction, and intercellular interactions (Jin et al., 2021; Reitsma et al., 2007; Tarbell and Ebong, 2008) (Figure 1B). These proteins and carbohydrates are proteoglycans, glycosaminoglycans (GAGs), and glycoproteins as shown in Figure 1B. GAGs are characterized by distinct disaccharide units repeats that give rise to different components such as heparan sulphate, chondroitin sulphate, hyaluronic acid, and different forms of sialic acids (Figure 1B), (Fu and Tarbell, 2013; Weinbaum et al., 2021). The functions of glycocalyx are very dependent on the way GAGs are arranged (Weinbaum et al., 2007; Choi and Lillicrap, 2020). Heparan sulfate expression patterns are dependent on endothelial cell activation and stimulation, which are shear rate dependent (Reitsma et al., 2007; Zeng et al., 2012). Primarily binding to syndecans-1, heparan sulfate makes up the majority of the GAGs, binding 3–5 heparan sulfate per syndecan with larger syndecans also binding chondroitin sulfate (Zeng et al., 2022) (Figure 1B). It is a linear sulphated polysaccharide, formed from 40–300 sugar residues, approximately 20–150 nm in length, and is anchored to the apical core proteins, syndecans and glypicans (Giantsos-Adams et al., 2013; Ebong et al., 2014). The syndecan family comprises of different members: syndecan-1,2,3 and 4. Syndecan-1 is reported to be present on the apical glycocalyx, whiles syndecan-4 is mostly found in the basal membrane of the endothelial cells. Not much is known about the structure and functions of syndecan-2 and syndecan-3, and merits further investigation (Koo et al., 2013). Members of the glypican family include six members glypican-1,2,3,4,5, and 6. Among these members of the glypican family, only glypican-1 is expressed on endothelial cell glycocalyx (Tarbell, 2010). Hyaluronic acid is a non-sulphated GAG that is not covalently bound to a core protein. Hyaluronic acid is usually much longer than protein attached GAGs (Weinbaum et al., 2003; Dogné and Flamion, 2020). Long chains of hyaluronic acid, attached to endothelial membrane bound receptors, such as CD44, are presumed to intertwine through the glycocalyx and provide part of its structure (Curry and Adamson, 2012), (Figure 1B). Sialic acid, attached to other core proteins via the terminal ends is another component of the glycocalyx (Mensah et al., 2019). The most prominent salic acid residues that are expressed on the endothelial cells includes ∝-2,6-linked, ∝-2,3-linked, and ∝-2,8-linked residues which interact with the cells through recognition binding (Betteridge et al., 2017; Mensah et al., 2019). The other endothelium components such as glycoproteins which include selectins, integrins and immunoglobulin-like molecules are known to interact with the endothelial glycocalyx (Rai et al., 2015; S Reitsma et al., 2007). P-selectin and E-selectin are usually upregulated during inflammation (Rai et al., 2015; S Reitsma et al., 2007).

The endothelial glycocalyx plays a crucial role in regulating endothelial cell permeability, inflammation, signal transduction, and anticoagulation, contributing significantly to vascular homeostasis. Serving as the vascular gatekeeper, the glycocalyx controls the passage of water, proteins, and various molecules (Butler et al., 2020). Studies, such as the one conducted by Van Haaren et al. (2003), using different sizes of dextran to investigate rat myocardial capillaries, indicate that the glycocalyx acts as a barrier, restricting the passage of specific molecules through its layer. Degradation of the glycocalyx, as observed in studies by Kang et al. (2021) and van Haaren et al. (2003), allows the transport of water and low-density lipids into rat abdominal aorta cell membrane.

The degradation of the glycocalyx is recognized as a key indicator of inflammation. When the glycocalyx undergoes degradation, it exposes vascular adhesion receptors, facilitating the binding of immune cells (Kang et al., 2021). Fatal diseases such as sepsis and COVID-19 involve a systemic breakdown of the glycocalyx, believed to be initiated by specific enzymes and cytokines targeting the glycosaminoglycans (GAGs) within the glycocalyx. These enzymes lead to instability, reducing both the overall thickness and coverage of the glycocalyx (Uchimido et al., 2019). It is noteworthy that the comprehensive impact of inflammation and its underlying mechanistic pathways on the glycocalyx remains incompletely understood, warranting further investigation.

The glycocalyx serves a crucial role beyond regulating permeability and contributing to inflammation—it acts as a mechanotransducer. According to Weinbaum et al. (2003), hydrodynamic forces acting on the apical side of the glycocalyx’s core proteins result in a bending moment that transduces fluid shear stress. The tension in GAGs induced by hydrodynamic drags, caused by blood flow through the glycocalyx layer, transmits fluid shear stress through the core proteins into the cell cytoskeleton (Weinbaum et al., 2007).

From a cellular perspective, the drag force experienced by the glycocalyx during blood flow in vessels translates mechanical forces through the core proteins into the cell cytoskeleton. Studies by Zeng and Tarbell (2014) delves into the dynamics of spatial redistribution of the actin cytoskeleton in response to shear stress. Under static conditions, dense peripheral actin bands were present at the cell periphery of rat fat pad endothelial cells. After 30 min of shear stress, noticeable polymerization and polarization of actin filaments occurred, with stress fibers oriented parallel to the nearest edge, and the emergence of lamellipodia and filopodia. Extended exposure to 24 h of shear stress further reinforced the polymerization and polarization of actin filaments, leading to the observation of prominent stress fibers (Zeng and Tarbell, 2014).

The glycocalyx is recognized for its anticoagulation properties, with components like proteoglycans and glycoproteins (Figure 1B) binding to anticoagulation mediators such as antithrombin III, heparin cofactor II, and thrombomodulin (Reitsma et al., 2007). Specifically, the interaction between antithrombin III and the heparan sulfate of the glycocalyx strengthens its anticoagulant properties. Additionally, thrombomodulin binds to the chondroitin sulfate of the glycocalyx, initiating specific anticoagulation pathways (Kozar and Pati, 2015). Diseases such as COVID-19, disseminated intravascular coagulation (DIC), sepsis, cancer and malaria are characterized by glycocalyx degradation (Chelazzi et al., 2015; Buijsers et al., 2020; Huang et al., 2021) and circulating levels of specific glycocalyx components such as syndecan, may be used as markers for endothelial dysfunction and diseases severity.

While endothelial extracellular proteins like von Willebrand factor and P-selectin are known to play a role in the inflammation and coagulation process, their direct interaction with the glycocalyx and the exact mechanism of participation remain unclear and is the focus of this review.

### 2.2 The von Willebrand Factor

The von Willebrand Factor is a glycoprotein that plays an integral role in the maintenance of hemostasis (Peyvandi et al., 2011). It is composed of repeating subunits that form a long multimer (Cortes and El-Nakeep, 2023). The von Willebrand Factor is named after Erik von Willebrand, a Finnish doctor who documented a family with a seemingly hereditary bleeding disease, now known as von Willebrand disease (VWD) (Peyvandi et al., 2011) and was able to differentiate this disorder from hemophilia, despite similar symptoms (Favaloro, 2014). In the early 1960s, the combined deficiency of Factor VIII and an unknown protein was recognized as the primary cause of VWD. With advancements in ristocetin testing in the 1970s, von Willebrand Factor’s role in VWD was confirmed (Kreuz, 2008; Favaloro, 2014). The synthesis of von Willebrand Factor primarily happens in endothelial cells (Jaffe et al., 1974) and megakaryocytes (Sporn et al., 1985).

von Willebrand Factor has different conformations depending on the magnitude of shear stress that the glycoprotein is exposed to (Okhota et al., 2020). The original form of von Willebrand Factor is very similar to a folded protein, existing in a globular state. Unlike a folded protein, von Willebrand Factor does not have a sequence specific binding protein (Schneider et al., 2007). Therefore, the monomers can orient themselves in any order and in the direction of the shear stress field (Di Stasio and De Cristofaro, 2010). The changes to the orientation and state of the glycoproteins take place at a very small length scale, changing about 10 nm at a time (Singh et al., 2009). von Willebrand Factor structure is advantageous for capturing and binding to platelets in the bulk flow, which is essential in the regulation of hemostasis (Di Stasio and De Cristofaro, 2010).

Changes in von Willebrand Factor are dependent on the shear rate present in the cellular microenvironment. At low shear rates (10–1,000/s) von Willebrand Factor remains in a compact conformation (Schneider et al., 2007). The von Willebrand Factor experiences minimal alterations at shear rates below 3,000/s, with the overall size remaining unchanged, exhibiting only modifications at the domain level (Singh et al., 2009). The unfolding of von Willebrand Factor begins in a condensed arrangement when shear stress levels reach 30 dyne/cm^2. As the glycoprotein unfolds, it stretches and adopts a chain-like conformation, aligning itself in the overall direction of the shear stress field (Di Stasio and De Cristofaro, 2010).

The role of von Willebrand Factor in hemostasis is spurred by its binding patterns to platelets and connective tissue (Peyvandi et al., 2011). The A1 domain holds the primary function of being the binding site for the platelet receptor protein, GP1b. Platelet aggregation occurs at injury sites, resulting in the introduction of other plasma proteins such as Factor VIII in addition to von Willebrand Factor leading to thrombi formation (Ruggeri et al., 2006; Zhang et al., 2009). von Willebrand Factor can exist in ultralong forms which are cleaved in a shear stress dependent manner by ADisintegrin and Metalloprotease with ThromboSpondin motif (ADAMTS13) to prevent unwanted coagulations (Zhang et al., 2009; Bartoli et al., 2015; Zheng, 2015). The anchorage of ultralong von Willebrand Factor to the endothelium is thought to be mediated by P-selectin expressed on the endothelial bed (Padilla et al., 2004), a mechanism that requires further investigation.

Under normal physiological conditions, the von Willebrand Factor will act according to the processes described above to successfully encourage platelet aggregation (Peyvandi et al., 2011), and the eventual cessation of bleeding. However, its function during a disease state is less concrete (Ladikou et al., 2020). Thrombotic incidents are prevalent amongst critically ill COVID-19 patients (Mei et al., 2021), an infection caused by SARS-Cov-2 virus (Li et al., 2020). This acute coagulation disorder is termed COVID-19-associated coagulopathy and the von Willebrand Factor potentially contributes to its development (Mei et al., 2021). Elevated levels of both von Willebrand Factor activity and antigens have been associated with clinically adverse outcomes for patients, leading to questions about its contribution to this pathophysiology. In addition to elevated von Willebrand Factor levels, decreased levels of the ADAMTS13 enzyme have been reported, meaning an increase amount of uncleaved von Willebrand Factor would be in circulating during this condition (Mei et al., 2021).

Von Willebrand Factor’s effects during COVID-19-associated coagulopathy does not appear unique to the virus, but rather to the presence of inflammation, as other conditions such as sepsis, DIC, cancer, and malaria also show marked effects on the von Willebrand Factor. Severe DIC outcomes are also associated with an increase in von Willebrand Factor levels and a decrease in ADAMTS13 (Habe et al., 2012). This same pattern is observed during the massive immune response responsible for the development of sepsis and septic shock (Feroz Azfar et al., 2017). The widespread inflammation and endothelial dysfunction in these conditions is indicative of the relationship between endothelial damage and thrombotic dysregulation (Patel et al., 2019).

### 2.3 P-selectin

P-selectin is expressed and produced on the endothelial bed and megakaryocytes and encoded by the SELP gene in humans. One of the numerous proteins attached to the glycocalyx is P-selectin (S Reitsma et al., 2007). This selectin is composed of an N terminal lectin domain, an epidermal growth factor, about nine repeating regulatory proteins, as well as transmembrane sections and a small intracytoplasmic end (Blann et al., 2003). P-selectin is stored in the Weibel–Palade bodies and can be localized in the ⍺-granules during exocytosis, bringing it to the cell surface when stimulated by thrombin. P-Selectin is expressed during endothelial activation and lasts for a short time and quickly internalized and destroyed within the cell (Merten and Thiagarajan, 2004; Reitsma et al., 2007; Tvaroška et al., 2020). P-selectin binds to heparan sulfate (Wang and Geng, 2003), using the P-selectin glycoprotein ligand 1 (PSGL-1) and the GP-IB-IX-V complex. The binding to the GP-IB-IX-V complex allows P-selectin to mediate platelet adhesion, furthermore, stabilizing the GPIIb/IIIa fibrin interaction mediates inter-platelet aggregation (Blann et al., 2003). P-selectin is important in the initial adhesion of platelets and leukocytes during injury and inflammation, hence plays a major role in homeostasis and thrombosis (Agrati et al., 2021). Conditions like COVID-19 which is known to cause the increased production of Tumor Necrosis Factor Alpha (TNF-alpha) results in the increased transcription of P-selectin (Tvaroška et al., 2020).

DIC and some cancers are pro-thrombotic events, manifesting as microthrombi events, the over expression of P-selectin promotes coagulation through the circulation of particles with its counter receptor, PSGL-1, that bring tissue factors to the platelet thrombus (Mosad et al., 2011; Agrati et al., 2021). The release of soluble P-selectin in the plasma of patients with DIC is reported to be an indicator for the severity of the disease (Wang et al., 2012). Other inflammatory associated disease such as sepsis is also associated with soluble P-selectin and the level of blood concentrations of P-selectin has been correlated with the severity of sepsis (Zonneveld et al., 2014). Expression of P-selectin can also be seen when the endothelium is damaged, activating the interactions between the leukocytes, platelets and endothelium. P-selectin’s role in inflammation and thrombotic events and location on the endothelium makes it an ideal biomarker for studying pro-thrombotic diseases and conditions (Perkins et al., 2019). While P-selectin is a great biomarker, the binding site is very short, it cannot be detected in healthy conditions and can only be discovered during glycocalyx degradation (Moore et al., 2021).

## 3 Inflammation-induced coagulation

Inflammation is the body’s physiological response to injury or infection (Wong, 2021). External symptoms of inflammation include swelling, pain and heat (Nathan and Ding, 2010; Ahmed, 2011). Underlying physiological changes induced by inflammation include vasodilation, edema and the migration of immune cells to the affected tissue (Nathan and Ding, 2010; Alessandri et al., 2013), (Figure 1A). Though inflammation typically responds to mitigate damage or contamination, the response sometimes causes additional risk (Wong, 2021). Certain pathogens have shown the ability to change the proinflammatory cytokine cascade into a cytokine storm that causes heightened inflammation and overproduction of proinflammatory cytokines (Fajgenbaum and June, 2020). Inflammation initiates coagulation, decreasing the effect of anticoagulants mechanisms and blocking fibrinolytic systems (Esmon, 2005, 2004). Cytokines are the mediators involved in coagulation activation resulting in endothelial cell dysfunction by causing the cells to be less responsive to inflammatory mediators (Esmon, 2005).

Uncontrolled coagulation is an effect of certain human pathologies. One defining factor in this phenomenon is the presence of an inflammatory immune response, primary to coagulation dysregulation (Wong, 2021). The underlying cause of this inflammation varies by disease state, ranging from infection to genetic autoimmune conditions (Del Carmen et al., 2018). One such pathology is COVID-19 caused by the coronavirus SARS-CoV-2, recently widespread in a global epidemic. This viral infection initiates a hypercoagulative state, although direct physiological pathways relating coronavirus infection, inflammation and coagulation are poorly understood (Colling and Kanthi, 2020). There are controversies in the literature as to whether endothelial cells are directly or indirectly affected by SARS-CoV-2 (Schimmel et al., 2021). It is known however, that the presence of the virus results in an influx of cytokines leading to widespread damage to vasculature (Colling and Kanthi, 2020). Patients with extreme cases of COVID-19 presents with uncontrolled coagulation referred to as COVID-19-Associated Coagulation, and the etiology of this pathology is still unclear. We believe that extreme damage to the endothelial glycocalyx during viral infection is critical for the onset and progression of coagulation during COVID-19 infection (Yamaoka-Tojo, 2020; Suzuki et al., 2021; Zhang et al., 2021; Yuan et al., 2022).

Another pathology that can lead to uncontrolled coagulation is DIC which results in overactive clotting leading to blood clots throughout the blood vessels (Okamoto et al., 2016). These clots can reduce or block blood flow and cause organ damage (Yamaoka-Tojo, 2020). As the pathology progresses the overactive clotting depletes blood platelets and clotting factors (Okamoto et al., 2016). DIC is usually caused by inflammation, resulting in excessive activation of Factor VII spurring fibrin and thrombin production disproportionately (Ryan and Costello, 2023). DIC can be caused by COVID-19 or similar infection, causing tissue Factors to be released from damaged endothelial cells in response to cytokines, released into the bloodstream (Ryan and Costello, 2023).

Sepsis, which is the most common risk factor for DIC, is also caused by uncontrolled inflammation. This condition arises from an infection within the body, leading to a hypercoagulative state and concurrent damage to tissue and organ. In the vascular system, perturbed endothelial cells and mononuclear cells produce proinflammatory cytokines that promotes coagulation (Levi et al., 1997; Okamoto et al., 2016; Song et al., 2017). Proteins expressed on these cells such as thrombin elicits the production of monocytes chemoattractant proteins one and interlukein-6, and interleukin-8 (Esmon, 2000; Maneta et al., 2023), leading to intravascular fibrin deposition (Souza et al., 2015).

Cancer patients also experience an increased affinity for thrombosis and concurrent venous inflammation (Setiawan et al., 2022). As an acquired thrombophilia, cancer inflammation in the microenvironment induces a pro-inflammatory response including the release of Tumor Necrosis Factor Alpha (TNF-alpha) and cytokines including interleukins 1a, 6, 17, and 18 (Setiawan et al., 2022). This inflammation is responsible for widespread endothelial cell damage and concurrent risk of thrombotic episodes.

Another pathology that is present in the activation of the endothelial cells is Plasmodium falciparum, which is a severe form of malaria (O’regan et al., 2016). This parasite attaches to the endothelium disrupting the pathogenic processes of the molecules adhered to the endothelium (Yipp et al., 2003). Moderate to severe cases of malaria present a significant increase in plasma von Willebrand antigen levels (O’Donnell et al., 2022). Patients with Palsmodium falciparum have decreased amounts of ADAMTS13, therefore have an increase in ultra-long VWF multimers. It has been hypothesized that the VWF may be involved in the pathogenesis of this parasitic malaria, but the mechanisms behind it are still being investigated (O’regan et al., 2016).

The common presentation of vascular damage in diseases like COVID-19, DIC, sepsis, cancer and malaria underscore the intricate relationship between inflammation and coagulation. The dysregulation of these pathways leads to a vicious cycle of vascular dysfunction, microthrombi formation, and organ damage. We suspect that the endothelial glycocalyx play a significant role in the onset and progression of these pathologies. The compromised integrity of the endothelial barrier leading to degradation of the glycocalyx contributes to leakage of fluids and proteins, exacerbating organ dysfunction. Understanding these shared mechanisms is crucial for developing targeted therapeutic strategies to mitigate the severe consequences of these complex disorders. Further research is essential to unravel the specific molecular and cellular interactions driving vascular damage, providing a foundation for more effective interventions and improved patient outcomes.

The interactions between the endothelial glycocalyx, von Willebrand factor, and P-selectin form a dynamic and intricate network that orchestrates key processes in vascular health and hemostasis. Delving into the crosstalk among these components could unravel a fascinating interplay that influences vascular integrity, platelet function, and inflammation.

## 4 The anchorage of von Willebrand Factor to the vascular bed, a proposed mechanism involving the endothelial glycocalyx and P-selectin

Components of the endothelial cell extracellular membrane, such as P-selectin, α vβ3-integrins, and heparan sulfate, have been proposed as possible attachment mechanisms for the ultralong von Willebrand Factor fibers (Kalagara et al., 2018; Wang et al., 2022). While there is binding between P-selectin and von Willebrand Factor, research has shown that this binding is negligible under physiological conditions of magnesium and calcium (Huang et al., 2009). Furthermore, the role of αvβ3-integrins has been brought into question because anchored von Willebrand Factor fibers are still observed in vβ3-integrin knockout mice (Chauhan et al., 2007). These observations have led to the proposition of other possible binding components that may be acting on von Willebrand Factor. One possible explanation is that negatively charged GAGs may have electrostatic interactions contributing to the attachment of von Willebrand Factor (De Ceunynck et al., 2013). Heparan sulfate, of the glycocalyx (S Reitsma et al., 2007) is hypothesized to be a relevant factor for von Willebrand Factor binding (Wang et al., 2022). The limited evidence available suggest that the endothelial glycocalyx could play a role in anchoring von Willebrand Factor to the endothelium (Kalagara et al., 2018), and that both syndecans and glypicans could play a significant role in this cellular mechanism. It is suggested that syndecans −1 clustering is associated with colocalization of von Willebrand Factor fibers and degradation of heparan sulfate is reported to reduce von Willebrand Factor binding to the endothelial bed (Kalagara et al., 2018).

Kalagara et al. (2018) provided experimental evidence that the glycocalyx plays a crucial role in tethering the von Willebrand Factor to the endothelial bed, which they confirmed in their study that the glycocalyx anchors the von Willebrand Factor (Kalagara et al., 2018). In this experiment, wheat germ agglutinin (WGA), which stains the entirety of the glycocalyx components, was used (Kalagara et al., 2018). Using GAG specific antibodies and enzymes to target specific components of the glycocalyx could help isolate GAGs that are directly involved in the anchorage of von Willebrand Factor to the vascular bed in addition to what was shown by Kalagara et al. (2018) In addition, varying the shear stress magnitude could provide relevant data on the importance of shear stress in the anchorage mechanism. Recently, Wang et al. (2022) showed that heparan sulfate is responsible for binding to von Willebrand Factor under exposure to blood circulating melanoma cells. They concluded that cancer cells with low heparan sulfate levels evade von Willebrand Factor recognition and are prone to metastasis (Wang et al., 2022). Further investigating involving other GAGs is necessary to clearly characterize the role of the glycocalyx in the anchorage of von Willebrand Factor to the endothelial bed. It could be possible that heparan sulfate is not the only GAG involved in this anchorage mechanism.

The role of the glycocalyx in von Willebrand Factor anchoring is especially relevant in the cases of inflammatory pathologies. In a healthy state, it could be possible that the glycocalyx components, and P-selectin may work together to anchor von Willebrand Factor as shown in Figure 1C. In inflammatory pathologies (Figure 1C), where the glycocalyx (Lipowsky et al., 2011) is known to be degraded and ADAMTS13 is deactivated or are expressed in low levels (Matsumoto et al., 2021) P-selectin could be the only remaining attachment point for von Willebrand Factor. This could promote the premature untethering of ultralong von Willebrand Factor from the vascular bed leading to uncontrolled platelet activation in the bulk flow (Figure 1C). This could be a potential contributing factor to the onset and progression of diseases such as COVID-19-Associated Coagulation.

Investigating the distinct glycocalyx GAGs responsible for offering structural support to P-selectin and facilitating the anchoring of von Willebrand Factor holds the potential to unveil valuable insights for upcoming research directions. This exploration could pave the way for tailored therapeutic approaches in addressing conditions such as COVID-19-Associated Coagulation and various inflammatory-related diseases.

## 5 Conclusion

Research specifically focusing on individual GAGs within the glycocalyx to pinpoint those facilitating the anchorage of von Willebrand Factor to the endothelium (see Figure 1D) has not been carried out to date. While it is critical to identify distinct GAG interactions, it is noteworthy that P-selectin exhibits affinities for heparan sulfate and, to a lesser extent, sialic acid—both GAGs present in the endothelial glycocalyx in a calcium-dependent manner (Koenig et al., 1998). Previous studies utilizing heparinase, an enzyme that cleaves heparan sulfate from the glycocalyx, have shown a significant reduction in P-selectin binding on cell surfaces. This suggests that, in addition to sialic acid derivatives, P-selectin may also bind to heparan sulfate-like proteoglycans, indicating a broader spectrum of interactions (Ma and Geng, 2000). The investigation into whether P-selectin collaborates with glycocalyx components in anchoring ultralarge von Willebrand Factor remains an area with limited research, holding potential for insights into coagulation mechanisms associated with specific inflammatory pathologies.
