# Supplementary material for: Deciphering the triad of endothelial glycocalyx, von Willebrand Factor, and P-selectin in inflammation-induced coagulation
Source: Front Cell Dev Biol. 2024 Apr 30;12:1372355. doi: 10.3389/fcell.2024.1372355 (PMC11091309; doi:10.3389/fcell.2024.1372355)
Supplement: Supplementary file 1 [file DataSheet1.docx]

Supplementary Material

# Supplementary Figures and Tables

It is important to transparently acknowledge that ChatGPT version 3.5 was employed to rephrase specific portions of the manuscript. These sections are distinctly marked with italicized text and red font in the manuscript. It is essential to note that ChatGPT was utilized solely for the purpose of rewriting and enhancing clarity, without engaging in fact-checking. In adherence to Frontier policy, screenshots of the original input provided to ChatGPT, and its responses are included in the supplementary section of the manuscript.

## Supplementary Figure 1


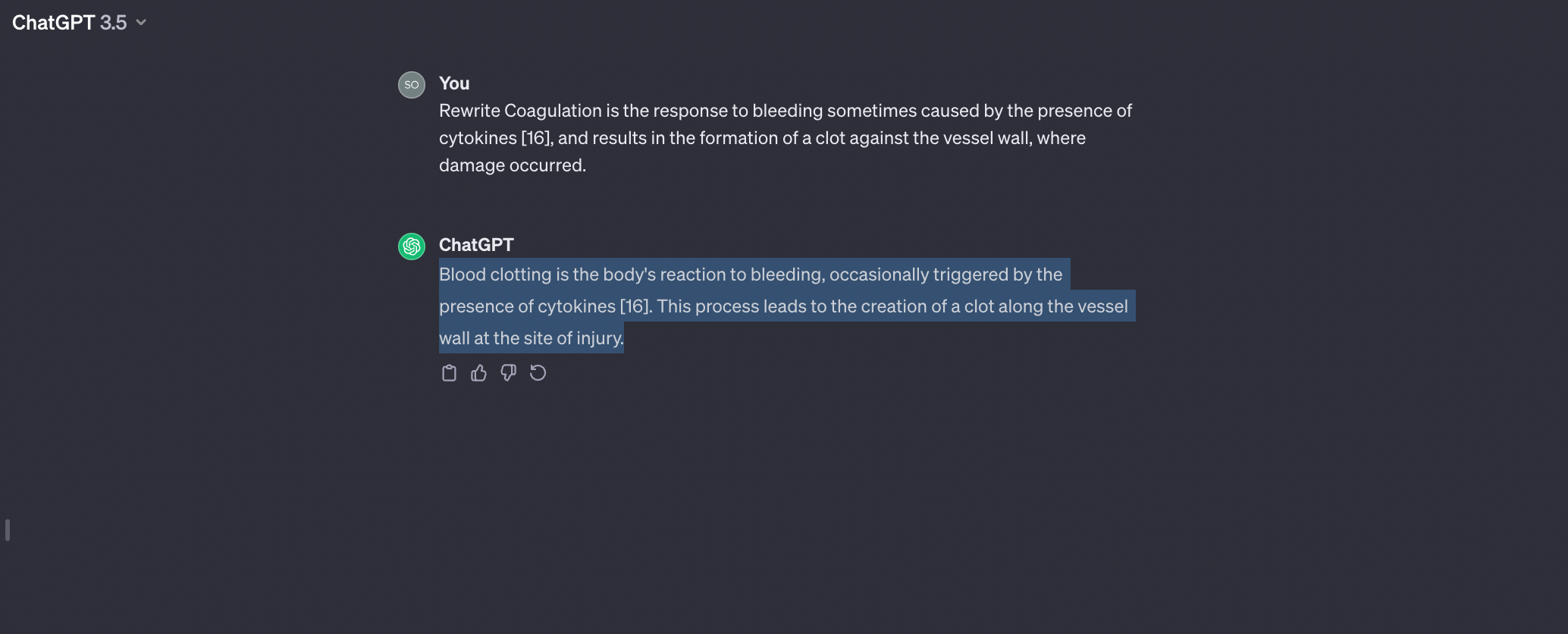


**Supplementary Figure 1.** Original text inputs and ChatGPT 3.5 outputs.

## Supplementary Figure 2


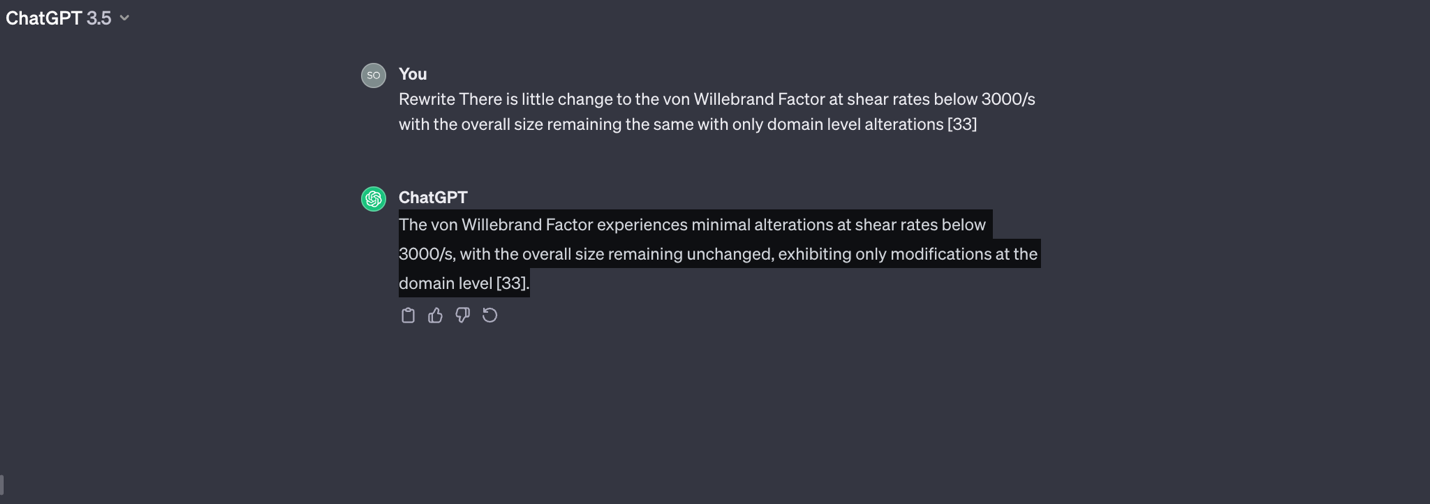


**Supplementary Figure 2.** Original text inputs and ChatGPT 3.5 outputs.

## Supplementary Figure 3


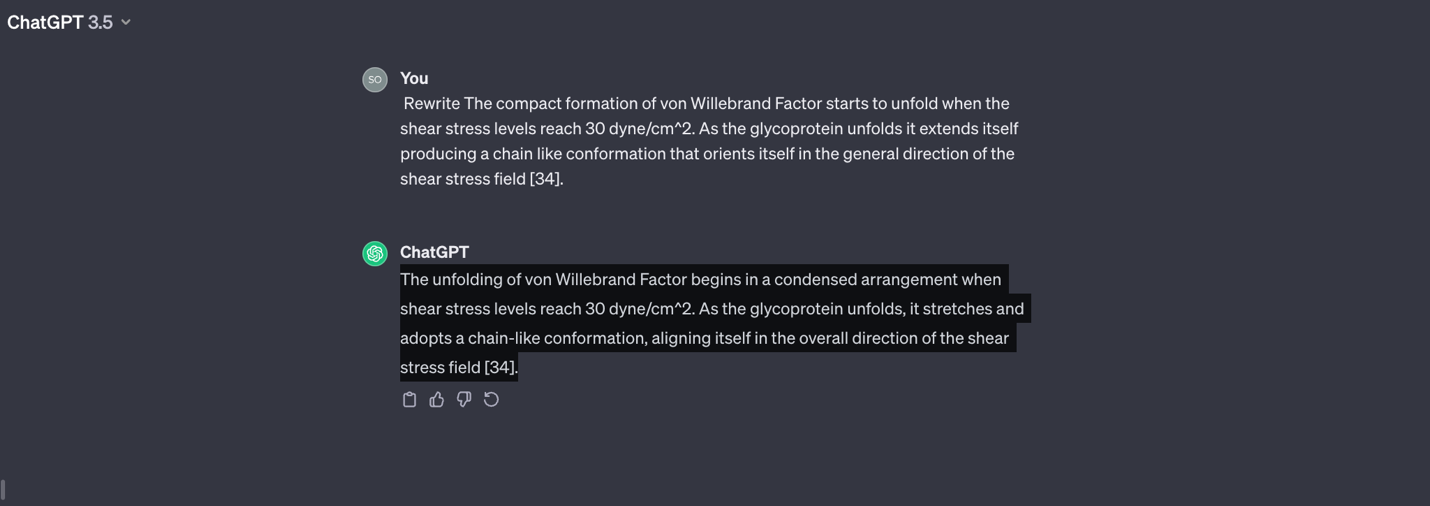


**Supplementary Figure 3.** Original text inputs and ChatGPT 3.5 outputs.

## Supplementary Figure 4


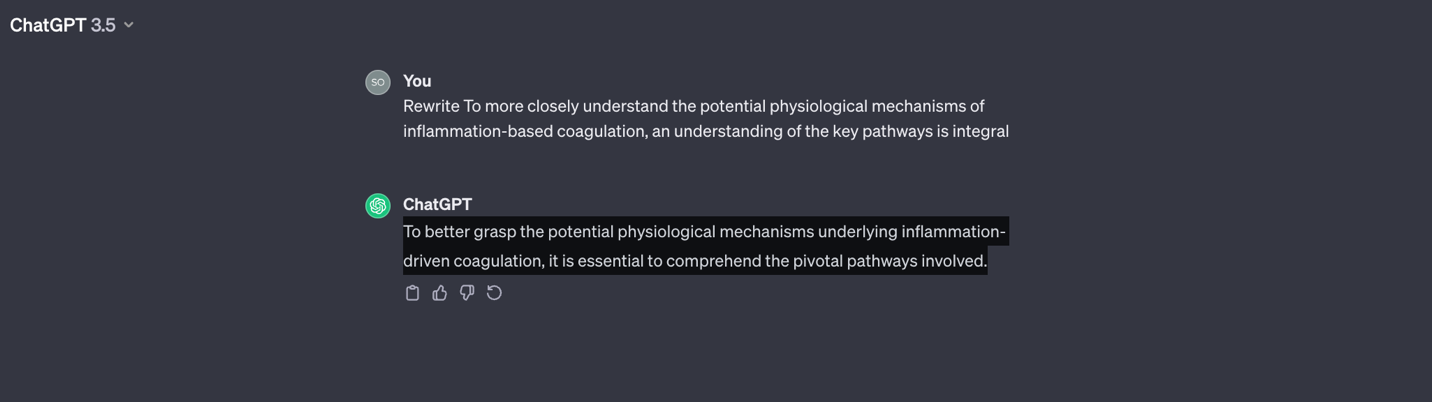


**Supplementary Figure 4.** Original text inputs and ChatGPT 3.5 outputs.

## Supplementary Figure 5


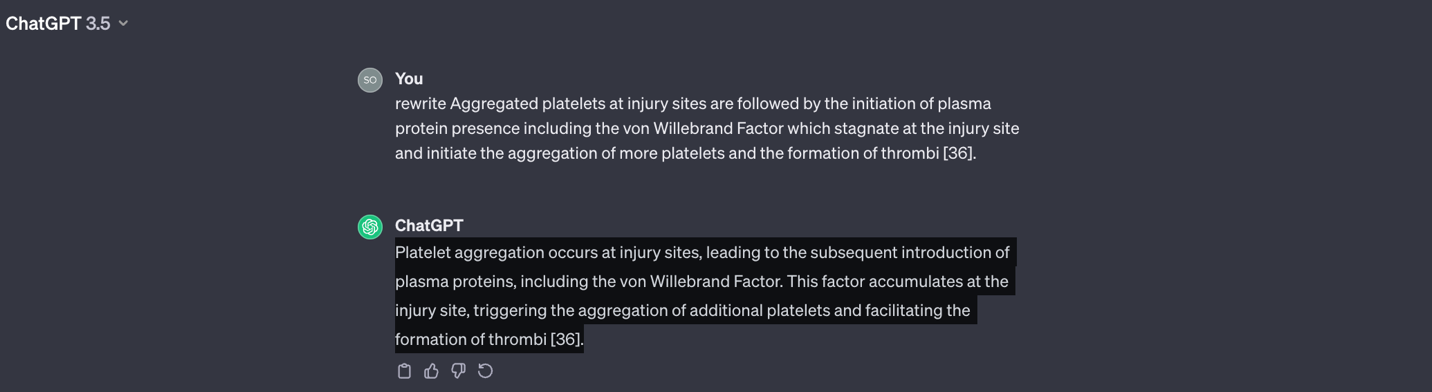


**Supplementary Figure 5.** Original text inputs and ChatGPT 3.5 outputs.

## Supplementary Figure 6


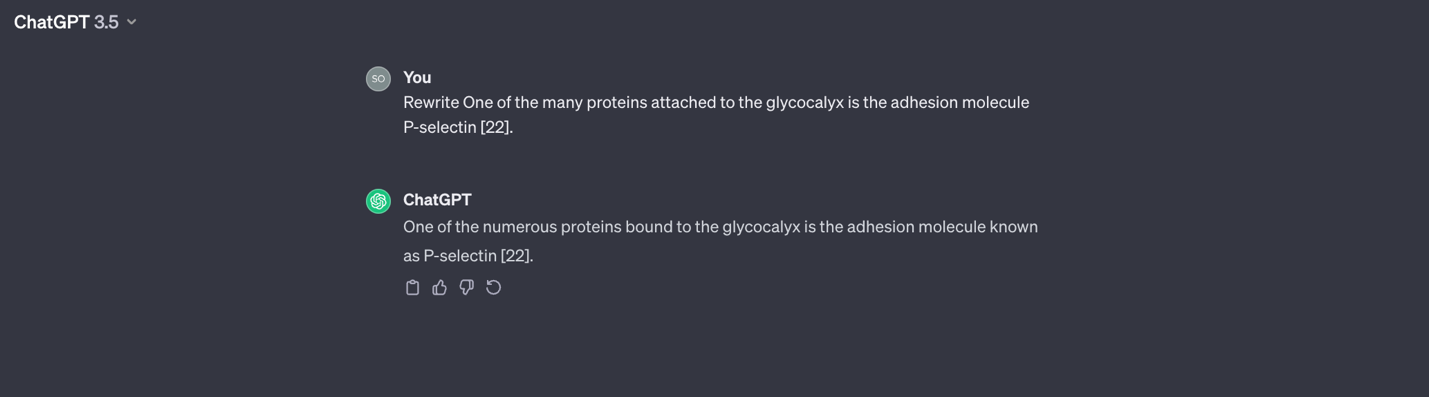


**Supplementary Figure 6.** Original text inputs and ChatGPT 3.5 outputs.

## Supplementary Figure 7


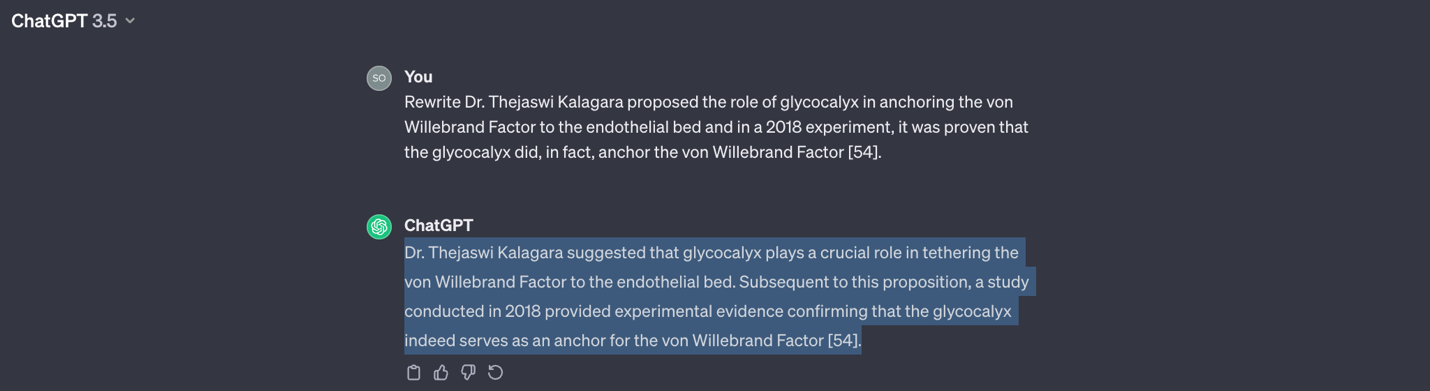


**Supplementary Figure 7.** Original text inputs and ChatGPT 3.5 outputs.

## Supplementary Figure 8


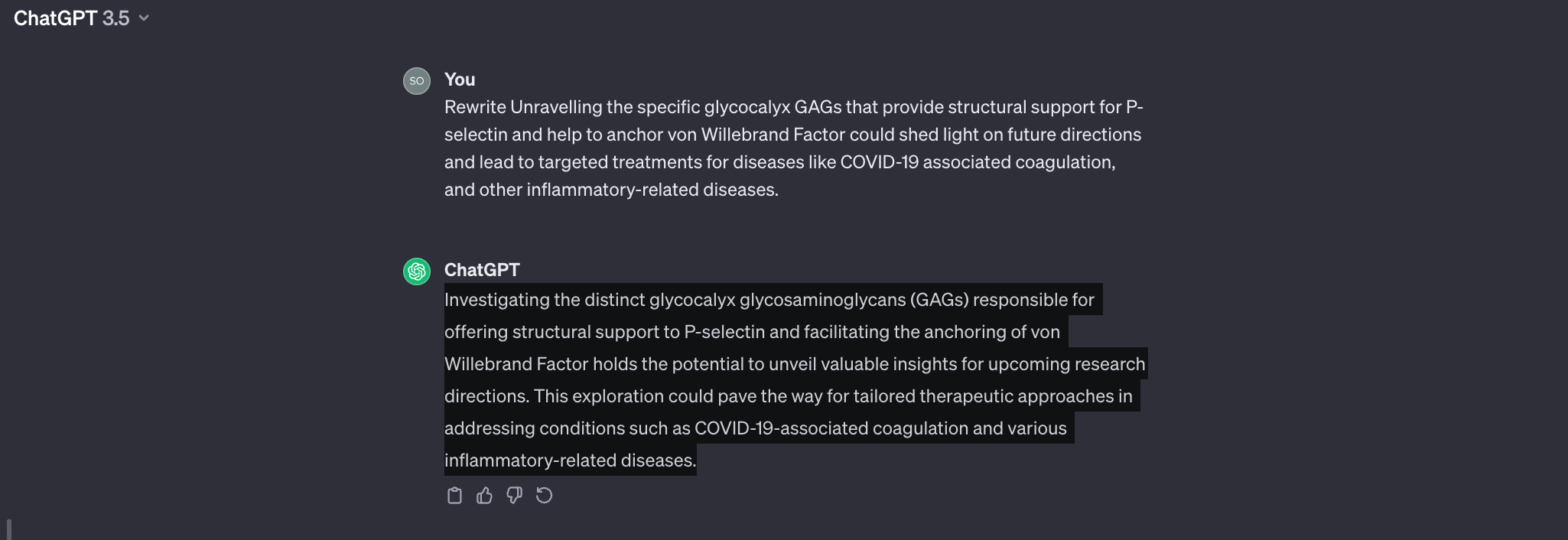


**Supplementary Figure 8.** Original text inputs and ChatGPT 3.5 outputs.

## Supplementary Figure 9


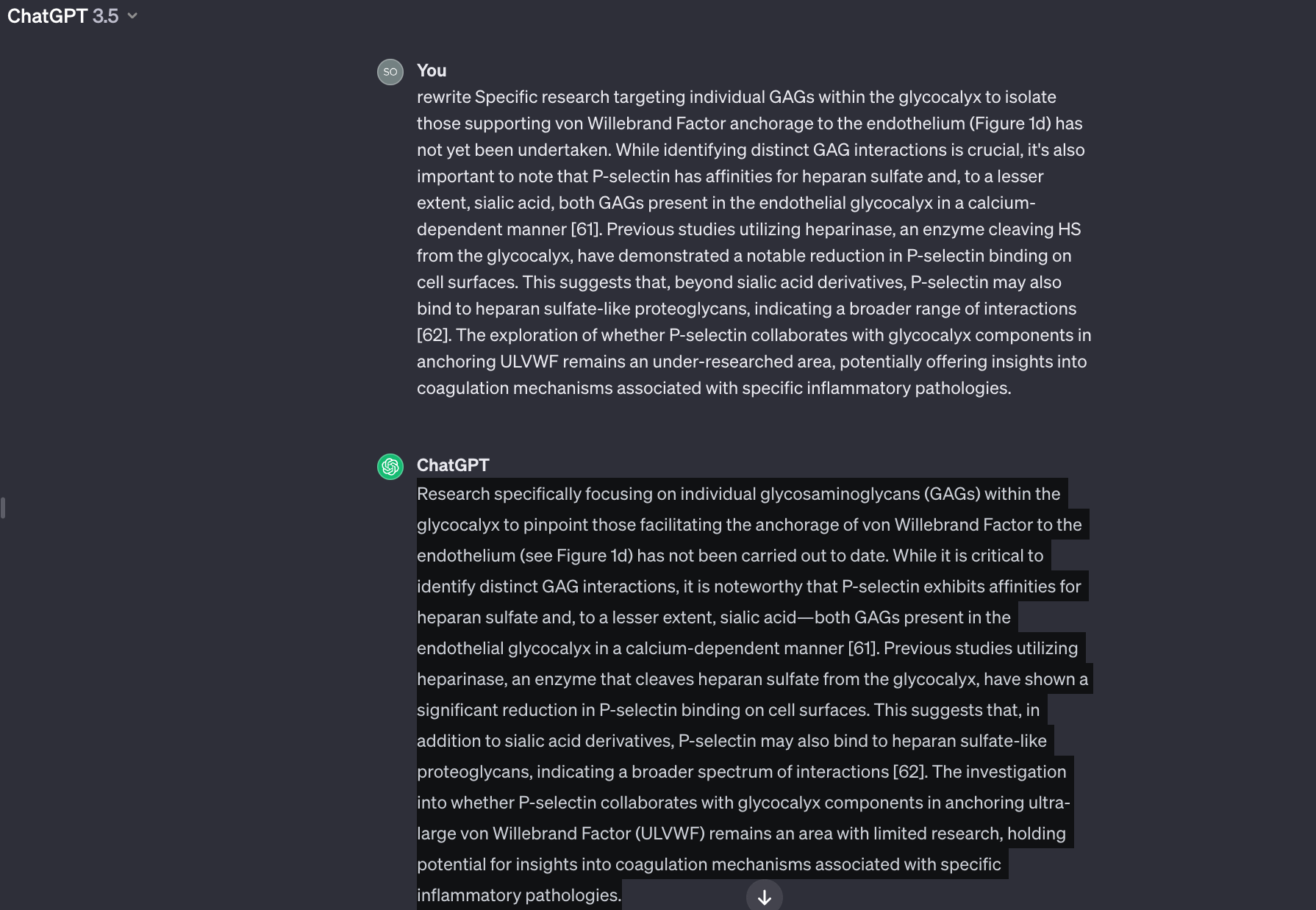


**Supplementary Figure 9.** Original text inputs and ChatGPT 3.5 outputs.

## Supplementary Figure 10


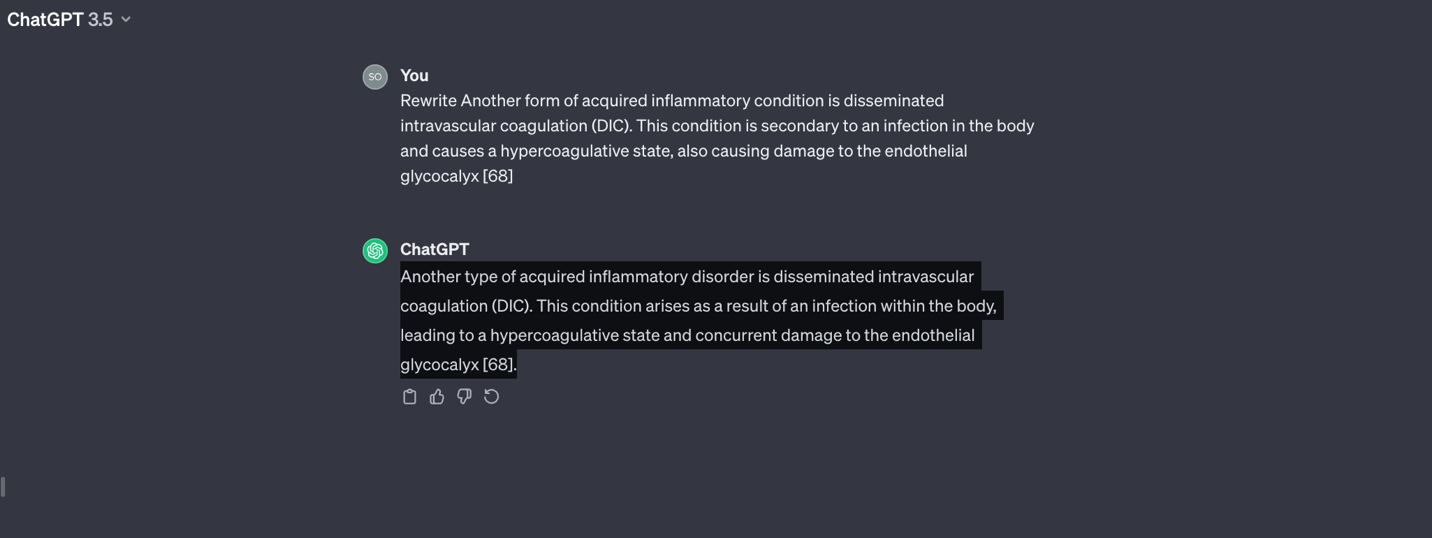


**Supplementary Figure 10.** Original text inputs and ChatGPT 3.5 outputs.

## Supplementary Figure 11


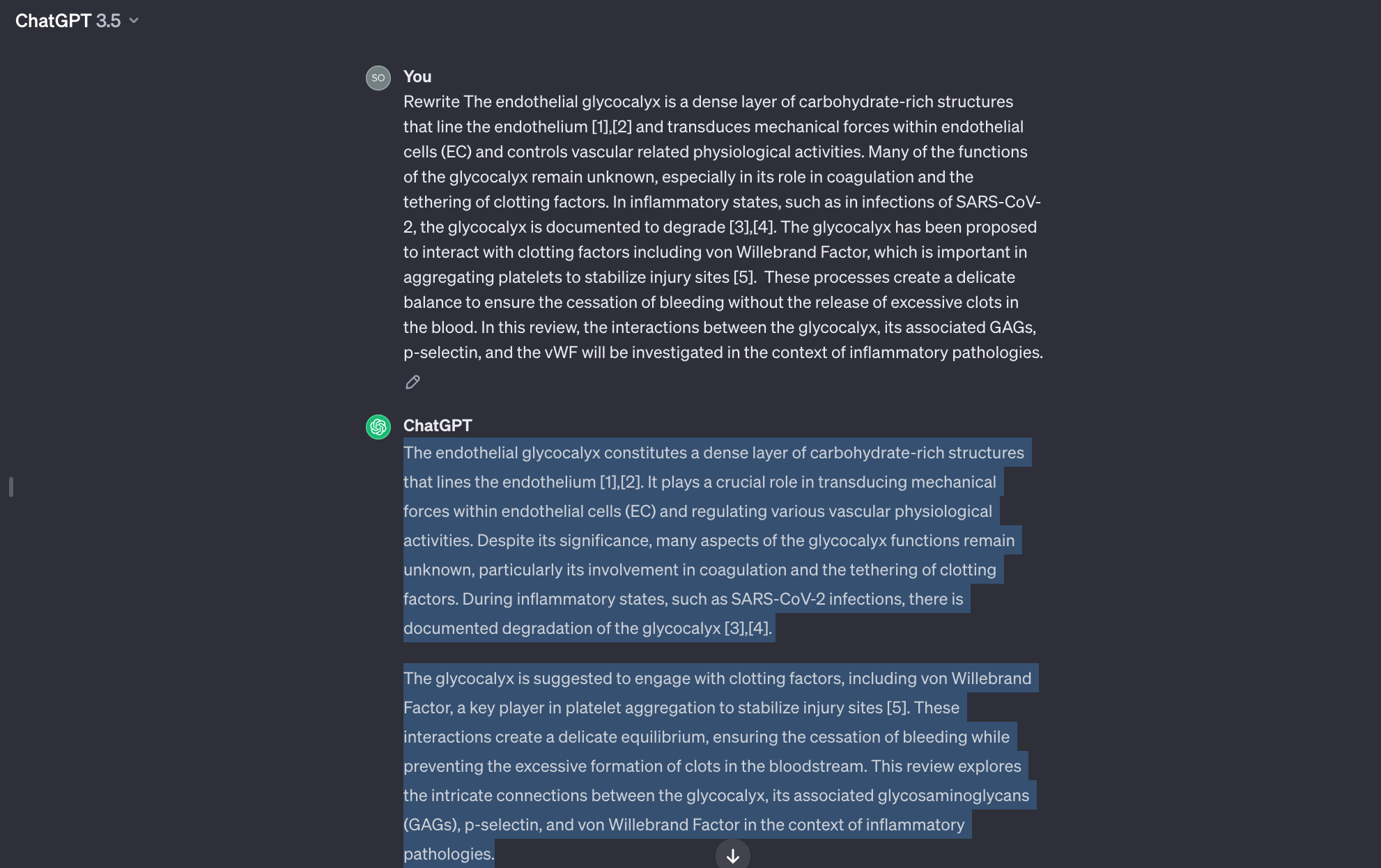


**Supplementary Figure 11.** Original text inputs and ChatGPT 3.5 outputs.
